# Does sadness bring myopia: an intertemporal choice experiment with college students

**DOI:** 10.3389/fpsyg.2024.1345951

**Published:** 2024-04-26

**Authors:** Peng Lei, Hao Zhang, Wenyu Zheng, Luoyi Zhang

**Affiliations:** ^1^China Center of Behavioral Economics and Finance, Southwestern University of Finance and Economics, Chengdu, China; ^2^School of Education and Psychology, Chengdu Normal University, Chengdu, China; ^3^School of Educational Sciences, Shangrao Normal University, Shangrao, China; ^4^Faculty of Humanities and Social Sciences, City University of Macau, Macau, China

**Keywords:** intertemporal choice, sadness, short-sighted behavior, logit regression analysis, moderating effect

## Abstract

**Introduction:**

While economics often interprets individual intertemporal choice preferences through the rationality assumption of utility maximization, the reality is that as emotional beings, individuals’ preferences for intertemporal behavior are much more diverse and inconsistent. Prior research has predominantly focused on positive or negative emotions based on prospect theory, such as anxiety, anger, disgust, and depression. However, there has been relatively little research on how sadness affects individuals’ preferences for immediate and future rewards.

**Methods:**

In this study, 170 college students are recruited as participants, and their emotions are primed with a video before engaging in an intertemporal task. Covariance analysis and logit regression model are established to examine the main and interactive effects of sadness on individuals’ immediate reward preferences.

**Results:**

The findings reveal that sadness led individuals to prefer smaller immediate rewards, demonstrating a more myopic behavioral pattern, but didn’t affect time discount rate. As the reward baseline increases, sadness’s impact on immediate reward preferences is more pronounced, exacerbating individuals’ myopic behavior.

**Discussion:**

In conclusion, these findings underscore the importance of considering emotional states in economic decision-making models and suggest avenues for future research to explore the complex dynamics of emotions and intertemporal choices.

## 1 Introduction

In daily life, individuals often face numerous decisions that involve trade-offs between the present and the future. For instance, they must decide whether to purchase a desired item immediately or wait for specific discount events in the future (e.g., Singles’ Day or Christmas) for potential savings. Other decisions might include more used for economic investments than current consumption, in order to guarantee larger consumption in future life, or deciding whether to immediately spend a received income or to invest it in a bank for future value appreciation. These temporal decisions pervade people’s lives, with a preference for immediate gratification often regarded as myopic behavior, while opting for larger future rewards implies a potentially stronger capacity for delayed gratification and, consequently, a greater likelihood of achieving higher accomplishments.

Although intertemporal choices are important and ever-present, people often make choices in a certain emotional state. Since the early 21st century, researchers have attempted to consider emotions as disruptive factors in decision-making to explore their mechanisms of influence on decisions. However, the research based on this does not fully explain the complex inter-temporal choice phenomenon. In order to better explain the individual’s decision-making behavior in intertemporal scenarios, the researchers began to pay attention to the impact of individual factors on their inter-temporal choices, such as uncertainty tolerance (Patak and Reynolds, 2007; Epper et al., 2011; Li et al., 2015), self-control impact on the propensity for inter-temporal choices (Kable and Glimcher, 2007; Luo et al., 2014; Bahrami and Borhani, 2023). At the same time, the impact of emotions on individuals’ intertemporal choice, as it belongs to both the susceptibility in individual factors and the stimuli in external factors, has long captured the attention of numerous scholars (Jiang and Sun, 2019; Dorison et al., 2020a; Dukes et al., 2021).

Research on incidental emotions’ impact on intertemporal choices has predominantly used a valence-based approach. Positive emotions lead individuals to overestimate positive outcomes and event likelihood, while underestimating negative outcomes and likelihood. In contrast, negative emotions have the opposite effect, making individuals less patient in intertemporal decision-making (Forgas, 1995; Desteno et al., 2000). This bias in negative emotional states, known as “motive transfer,” prioritizes immediate desires over long-term goals (Herman et al., 2003). Compared to a control group, individuals experiencing pleasure exhibit lower time discount rates, while those in negative emotional states show higher rates (Wang and Liu, 2009). Positive emotions increase patience, reducing time discounting and favoring long-term options, while negative emotions weaken patience, resulting in higher discounting rates and a preference for short-term options (Gray, 2004; Ifcher and Zarghamee, 2011; Molouki et al., 2019; Dorison et al., 2022).

Emotions, as posited by the Construal Level Theory (CLT), have an impact on the sensitivity to temporal distance by influencing cognitive construal levels. CLT suggests that individuals mentally represent events or objects at varying levels of construal, namely high-level and abstract or low-level and concrete, depending on the perceived psychological distance, which can encompass temporal, spatial, social, or hypothetical dimensions (Trope and Liberman, 2003). CLT asserts that distant future events are typically represented with abstract and essential features, indicating a high-level construal, while near future events are characterized by specific details, reflecting a low-level construal (Stillman et al., 2017). High-level construal tends to be more abstract and less concrete, while low-level construal tends to be more concrete and specific. In situations where events or objects are perceived as psychologically distant, individuals tend to adopt a high-level construal, resulting in more abstract and less concrete mental representations. This high-level construal is associated with a diminished emphasis on specific details and features, which can influence the perception of emotional valence.

When individuals adopt a high-level construal, they focus more on the overall emotional tone or the valence of an event or object, rather than specific sensory or perceptual details. This tendency can amplify the influence of emotional valence on judgments, evaluations, and decision-making processes. Errors of prediction, overconfidence, and underestimation of completion time can result from people’s failure to incorporate no schematic aspects of reality into their construal of future situations (Trope and Liberman, 2000). Emotions can also lead to judgment or decision biases through different appraisal tendencies and construal level mindsets (Han et al., 2014). Specifically, Negative emotions often lead to impatience and impulsivity, while positive emotions enhance patience by elevating cognitive construal levels (Rounds et al., 2007; Ifcher and Zarghamee, 2011). These valence-based perspectives find robust support in experimental studies (Elster, 1998; Jin and Isen, 2011; Lempert et al., 2015; Dorison et al., 2022), providing a solid foundation to understand emotions’ influence on intertemporal choice.

Specific emotion theories, such as the Appraisal Tendency Framework (ATF), argue against solely categorizing emotions based on valence levels when exploring their impact on intertemporal choice (Lerner and Keltner, 2000). According to the ATF, each emotion comprises multiple cognitive appraisal dimensions, with different dominant dimensions for each emotion, leading to distinct core appraisal themes. For example, sadness relates to definite losses, while anxiety relates to uncertain dangers. Extending the ATF, research has found that two negative emotions, anger and fear, have contrasting effects on risk perception (Lerner and Keltner, 2001). Experimental studies further demonstrate that classifying the impact of emotions on decision-making solely based on valence has limitations, as different emotions have distinct effects on decision-making (Lerner et al., 2003, 2013; Ferrer and Ellis, 2019). Within the ATF, each specific emotion is characterized by dominant cognitive appraisal dimensions, forming specific appraisal tendencies (Lerner et al., 2004). These tendencies can influence decision-makers’ information processing content, depth, or styles over time, resulting in varying effects on judgments and decisions. In summary, these studies suggest that the effects of emotional valence on intertemporal choices are inconsistent, and specific emotions of the same valence may have different effects on intertemporal choices.

How does sadness influence individuals’ attitudes toward the present and the future? Sadness itself implies a sense of loss (Lazarus, 1991), and in terms of the valence of sadness, there exists a certain heterogeneity. According to CLT, emotions play a role in shaping cognitive construal levels, which in turn affect decision-making. Sadness is generally associated with a more concrete and low-level construal. When individuals experience sadness, their attention tends to be focused on immediate concerns and specific details, rather than abstract and future-oriented considerations. This heightened focus on the present can impact individuals’ intertemporal choices. While according to the Appraisal Tendency Framework (ATF), sadness is classified as a low-certainty emotion. When individuals experience sadness, their internal uncertainty increases, leading them to attempt to reduce the uncertainty in their environment (Lerner et al., 2015). Research by Small and Lerner has demonstrated the influence of certainty levels. When participants’ information processing abilities were not restricted, those in a state of sadness were found to be more supportive of favorable welfare policies compared to individuals in a neutral or angry state (Small and Lerner, 2008). Lerner et al. explored the relationship between sadness and disgust, two emotions within the ATF, and economic value estimation in the context of consumer decisions. They found that sadness is associated with experiencing unavoidable losses, while disgust arises from approaching unacceptable things or viewpoints (Lerner et al., 2013). Consequently, in economic value estimation, individuals experiencing sadness may seek to modify their current environment to avoid the experience of loss, while individuals experiencing disgust may exhibit avoidance tendencies to reduce their disgust.

Furthermore, sadness, as a negative emotion, can also lead to increased impatience and a preference for immediate rewards in intertemporal decision-making (Lerner et al., 2013). However, it’s important to note that the relationship between sadness and impatience is not always straightforward and can be influenced by various factors. The intensity of sadness, individual differences, and contextual factors may moderate this relationship. For example, as the intensity of sadness increases, individuals may become more focused on avoiding negative experiences in the present, which could lead to a shift toward future-oriented choices and increased patience (Zhou et al., 2022). This inconsistency leaves room for continued exploration in this study’s qualitative and quantitative explanations of the role of sadness in intertemporal choice.

Additionally, the arousal of sadness is not a singular experience; it is often accompanied by various other emotional experiences, such as disgust, anger, and depression. These co-occurring emotions also synergistically impact intertemporal behavior in sad contexts, making the influence of sadness on time preferences more complex. In comparison to emotions like anxiety, depression, and anger, the research on the impact of sadness on intertemporal choice is relatively limited, and most studies highlight differences through a comparative approach with other emotions (Ferrer et al., 2016; Suo et al., 2021). There is scant focus on exploring intertemporal behavior variations within the context of sadness itself. To our knowledge, many studies investigated the impact of incidental emotion on intertemporal choice by adopting a between-subjects design, in which participants were induced to a specific enduring mood state by reading autobiographical stories, watching film clips, or conducting different cognitive tasks (Wang and Liu, 2009; Hirsh et al., 2010; Raeva et al., 2010; Li and Xie, 2012; Lerner et al., 2013).

Except emotion, individual’s intertemporal choice is also influenced by other underlying factors, such as the motivational rewards involved in the decision-making, which significantly impact an individual’s choice between immediate and future benefits (Green et al., 1997). Higher reward amounts indicate greater absolute changes in future benefits due to the delay in intertemporal choices. Although individuals may be aware of the constant discount rate and the same rate of change, the utility gained is not linear. The length of the intertemporal period faced by individuals is also a crucial factor affecting their intertemporal choices. If a longer period does not bring significantly higher returns, individuals’ choices tend to favor obtaining more certain immediate gains, resulting in reduced patience toward future benefits (Takahashi et al., 2008a,b). Demographic variables such as age, gender, income level, education level, and cultural background also have different effects on intertemporal choice. Generally, males tend to have stronger patience toward future benefits compared to females (Keidel et al., 2021), and older individuals tend to exhibit greater patience toward future benefits compared to younger individuals (Seaman et al., 2022). For different income groups, the relative level of incentives and income plays a crucial role in influencing intertemporal choice. If the relative difference is too small, individuals may prefer immediate benefits, whereas if it is significant, they may opt for future benefits. Lastly, individual intertemporal choice also varies across different cultural backgrounds. Western cultures tend to exhibit a preference for immediate benefits compared to Eastern cultures, which emphasize waiting and anticipation (Takahashi et al., 2010).

When considering the impact of sadness on individuals’ intertemporal choice, it is essential to also take into account the potential moderating factors. Previous research has examined the moderating effects of incentive amounts and intertemporal lengths due to their practical manipulability and relevance. In addition to focusing on the main effect of sadness, our study investigates the moderating effects of other factors on the influence of sadness on intertemporal choice. By doing so, we aim to gain a more comprehensive insight into the relationship between sadness and intertemporal choice. Therefore, to explore the impact of sadness induction on individuals’ intertemporal choice from a more comprehensive perspective and to examine potential interaction effects with other variables, this study proposes the following research hypotheses:

*Hypothesis 1*: Sadness induction will significantly enhance individuals' preference for immediate rewards, leading to a manifestation of more short-sighted behavior.

*Hypothesis 2*: The interplay between baseline rewards and sadness induction is expected to exert a substantial influence on participants' current preferences in intertemporal choice. Specifically, the magnitude and direction of the impact of sadness induction on preference for immediate rewards will be contingent upon the level of baseline rewards.

*Hypothesis 3*: Anticipating a significant interplay between intertemporal lengths and sadness induction, we posit that this interaction will shape participants' current preferences in intertemporal choice. The influence of sadness induction on preference for immediate rewards will vary across different intertemporal lengths.

*Hypothesis 4*: The interaction between task difficulty and sadness induction is predicted to significantly affect participants' current preferences in intertemporal choice. The effect of sadness induction on preference for immediate rewards will be contingent upon the level of task difficulty.

## 2 Materials and methods

### 2.1 Experimental design

This study employs a single-factor completely randomized design, with the independent variable being the type of emotion induction (neutral and sadness). The main control variables include the gender of participants, baseline rewards, task difficulty, and intertemporal lengths. The dependent variable is participants’ preference in intertemporal choice, and the measurement indicator is the binary outcome of participants’ intertemporal choices (choice today or future days).

### 2.2 Participants

Based on the two group mixed design, we use Gpower software and set the effect size as 0.3, α as 0.05 and power as 0.95, then calculate the minimum subject requirement is 134. The participants are undergraduate students from Chengdu Normal University and other universities nearby through online and offline poster, with a total of 170 participants randomly assigned to the sadness group (43 female in 81 subjects) and the neutral control group (49 female in 89 subjects). Their average age is 19.65 ± 1.27 years old, 92 are female. The major background mainly includes education, management, etc., and the grade distribution is the first year and the second year. All participants are considered valid samples, and they are all in good physical and mental health, with normal or corrected-to-normal vision.

### 2.3 Experimental materials and instruments

In this experimental study, the effectiveness of emotion induction is a critical factor for the success of the experiment. Gross pointed out that film clips are a common form of entertainment that can be used in a relatively normal context, without deception and with easy manipulation, making them a suitable method for inducing emotions (Gross and Levenson, 1995). Additionally, a meta-analysis concluded 250 studies showed that video induction can successfully induce corresponding emotions (Gerrards-Hesse et al., 2011). Therefore, in this study, specific emotions are induced through video clips. In the emotion induction phase, participants in the sadness group watch a family-themed film clip titled “My Brothers and Sisters,” which lasts 3 min and 27 s, the material can effectively induce sadness in the subjects, and previous studies have proven that the sad state is in a generally elevated state and is significantly higher than the baseline level (Fang et al., 2009). While participants in the neutral group watch a documentary film clip titled “March of the Penguins.” which lasts 3 min and 30 s. The efficacy of the selected clips has been previously validated (Jin et al., 2009). The emotion self-assessment scale requires participants to report the intensity of 19 emotional states, including sadness (gloomy, disheartened, pessimistic) and neutral (numb, indifferent, unconcerned). Additionally, the scale includes other emotions such as fear, anxiety, anger, tension, amusement, boredom, pleasure, disgust, rage, happiness, frustration, and aversion (scale reference: Lerner et al., 2013).

**Figure 1:**
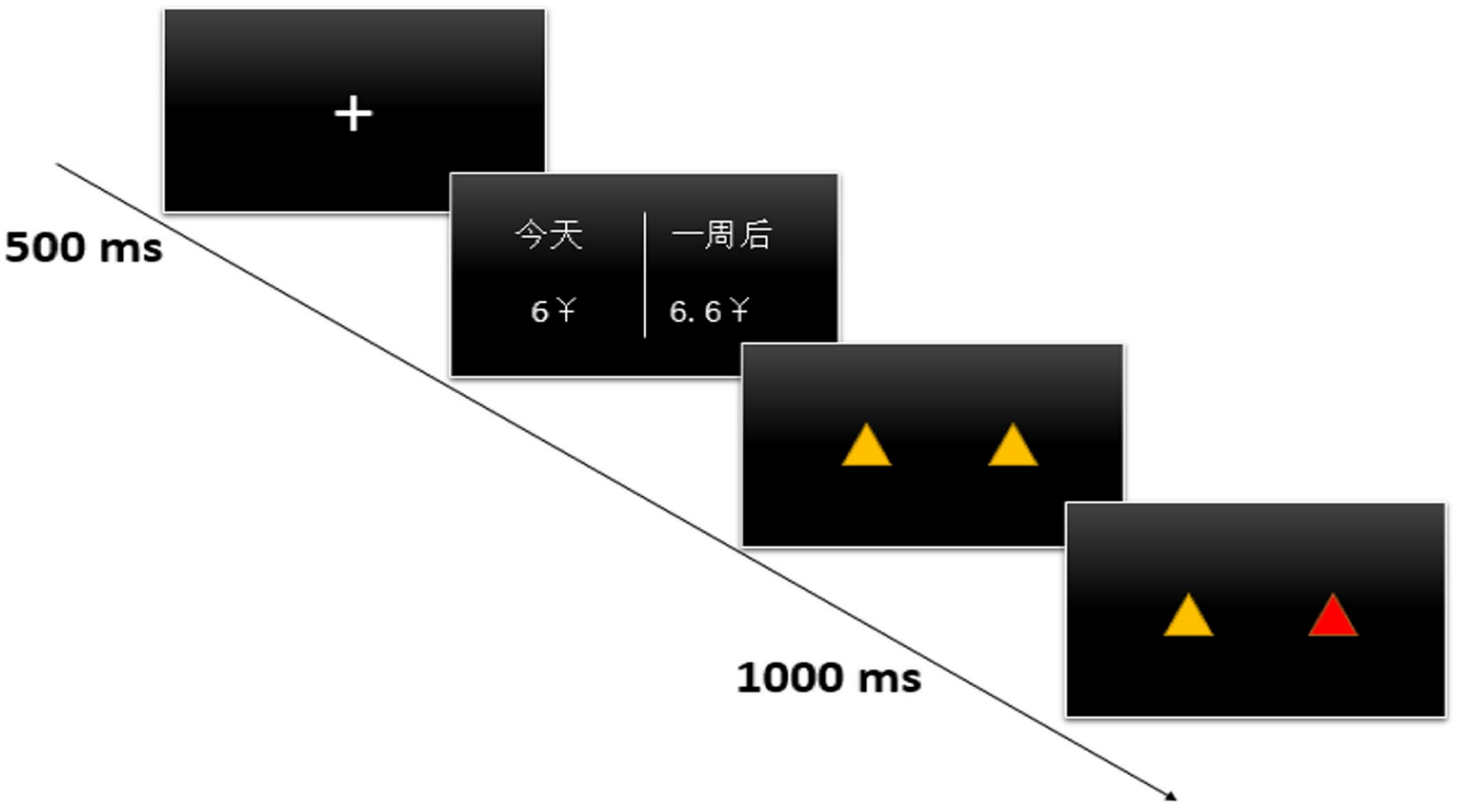
Each trial flowchart. This figure shows the process of each trail after the participants completed the video viewing task in the experiment, including the cross of 500 ms to focus the participants’ attention at the beginning, and then the intertemporal decision options of unlimited time. After the participants finished the choice, the red and yellow triangles of 1,000 ms would be presented to indicate the location of the choice.

In the intertemporal choice phase, the rate of monetary difference between alternative options (i.e., difference rate, (¥R’- ¥R)/¥R) is set at: 5, 10, 15, 25, 35, 50, 70, and 95%. Here, ¥R represents the smaller amount available for immediate redemption, and ¥R’ represents the larger amount available for redemption at a specific future time. In this study, baseline money ¥R contained two level, 2 RMB and 6 RMB. Following McClure et al.’s (2004) study, task difficulty is defined based on the variability in participants’ preferences when making choices between options with different percentage differences in dollar amounts. Choices where participants consistently selected the earlier reward or consistently chose the later, larger amount were categorized as “easy.” Conversely, choices with significant variability in preference were labeled as “difficult.” To further validate the categorization, we compared the reaction times between the two categories. Longer response times were interpreted as indicative of prolonged decision-making processes for the difficult choices. The temporal intervals option was constructed using one of two delays (3 and 7 days). The stimulus program is created and presented using the E-prime 2.0 software on a 14-inch LCD computer screen (Figure 1).

### 2.4 Experimental procedure

During the experiment, each participant sits comfortably in a quiet laboratory environment in front of a computer. The eyes are approximately 50 cm away from the computer display, with a white background color. The experiment consists of three stages: emotion induction stage, button-press practice stage, and formal test stage. Both groups of participants have the same tasks in the button-press practice stage and formal test stage, with the only difference being the tasks in the emotion induction stage.

Emotion Induction Stage: Participants first complete the emotion self-assessment scale as a pre-test of their emotions. All participants are randomly assigned to either the sadness group or the neutral control group. After watching the videos, participants complete the emotion self-assessment scale again as a post-test of their emotions. Immediately after completing the scale, participants proceed to the intertemporal choice task.

Button-Press Practice and Formal Test Stage: Before the formal experiment, participants undergo a practice session consisting of 20 trials. The practice procedure is identical to the formal procedure, and during the practice, the experimenter provides detailed instructions until participants become proficient in the task. As shown in the figure below, in each trial, a “+” sign appears in the center of the computer screen for 500 ms to signal the start of the experiment. Subsequently, two options appear on the screen, with the left option representing the immediate choice and the right option representing the delayed choice. Participants are required to make their choices based on their genuine thoughts as quickly as possible. They press the F button for the left option and the J button for the right option. After pressing the button, the triangle will change from yellow to red for 1,000 ms to confirm the choice. The next trial then follows.

The formal experiment consists of a total of 128 trials presented in random order. There are 8 trials for each money difference level, with an equal number of trials for each time interval. The experiment is divided into 4 blocks, each containing 32 trials, with self-controlled rest periods between adjacent blocks. Finally, the incentive method used in the experiment involves randomly selecting 5 intertemporal choices made by each participant, and the sum of these choices determines the participant’s final earnings. The immediate payments are made after the experiment, while the future payments are made promptly after the specified future time. On average, each participant receives a payment of 17.6 (±6.27) yuan. After experimental data collected, we use SPSS25 to finish the following analysis.

## 3 Results

### 3.1 Emotion induction effectiveness test

Following the approach of Lerner et al. (2013), the 19 emotional states are classified into six emotions: sadness, happiness, neutrality, fear, disgust, and anger. The scores for each emotion word are summed and totaled. Mean comparisons are conducted for the data before and after the induction of sadness emotion, and the results are shown in Table 1.

**Table 1:** Comparison of emotional intensity of sad group before and after watching video (*N* = 81).

Emotion type	Before watching		After watching		*t*	*p*
	*M*	SD	*M*	SD		
Sadness	6.63	3.77	10.05	3.60	−5.92	0.001
Happiness	13.68	3.42	7.90	4.63	9.07	0.001
Fear	7.16	3.65	6.50	3.30	1.21	0.228
Neutrality	11.36	4.00	8.35	3.90	4.85	0.001
Disgust	5.25	2.58	4.55	2.47	1.77	0.079
Anger	5.88	3.15	5.29	3.05	1.20	0.231

From Table 1, it can be observed that after watching the video, participants in the sadness group report significantly higher levels of sadness emotion compared to before (mean = 10.049 > 6.630, *p* < 0.001). Correspondingly, levels of happiness emotion significantly decrease (mean = 7.902 < 13.679, *p* < 0.001), as well as neutrality emotion (mean = 8.354 < 11.358, *p* < 0.001). However, there are no significant differences in the changes of other emotions (fear, disgust, anger, etc.). These data indicate that the induction of sadness emotion is effective and in line with normal psychological responses. As for the neutral emotion control group, the emotional data before and after watching the video shows no significant changes.

Furthermore, a comparative analysis is conducted between the sadness group and the neutral control group after watching the video, and the results are shown in Table 2.

**Table 2:** Comparison of emotions between sad group and neutral control group (*N* = 170).

Emotion type	Watch a clip from my brothers and sisters		Watch a clip from March of the penguins		*t*	*p*
	*M*	SD	*M*	SD		
Sadness	10.05	3.60	4.75	2.43	11.36	0.001
Happiness	7.90	4.63	11.70	3.58	−6.02	0.001
Fear	6.50	3.30	4.94	2.90	3.28	0.001
Neutrality	8.35	3.90	10.63	4.72	−3.42	0.001
Disgust	4.55	2.47	4.05	1.89	1.51	0.134
Anger	5.29	3.05	3.94	1.71	3.61	0.001

From Table 2, it can be observed that there is a significant difference in the reported levels of sadness emotion between the sadness group and the neutral control group (mean = 10.049 > 4.753, *p* < 0.01). Additionally, the happiness emotion reported by the sadness group is significantly lower than that of the neutral group. Participants in the sadness group also report relatively higher levels of fear and anger emotions compared to the neutral control group, but the difference in sadness emotion remains the most significant. Therefore, based on the above tests, it can be concluded that the induction effect of sadness emotion is significant.

### 3.2 Intertemporal choice result analysis of covariance

The covariance analysis (ANCOVA) was conducted to examine the influence of emotion induction, task difficulty, gender, and intertemporal length on participants’ intertemporal choice behavior. As previously mentioned, the calculation of task difficulty is based on the proportion of choices favoring immediate rewards in tasks with small differences between immediate and future rewards, or the proportion of choices favoring future rewards in tasks with large differences. In this study, choices with differences between 5% or 10 and 70% or 95% were classified as easy, while those with differences between 15 and 50% were considered difficult (refer to Table A1 for the specific percentages of delayed choices under each delay amount). In contrast to the categorization by McClure et al. (2004), this study utilized different ranges for easy and difficult tasks. To validate the appropriateness of this classification, a difference test was conducted on the reaction times of participants under different difficulty levels (RT_easy vs. RT_difficult = 1.92 s vs. 2.53 s, *p* = 0.012). The results indicated that the average reaction time was shorter for easy tasks, supporting the validity of this classification.

The covariance analysis results unveiled several significant main effects and interaction effects, shedding light on the intricate dynamics of decision-making processes, see as Table 3. The overall model exhibited significance [*F* (9, 21,750) = 171.214, *p* < 0.001], indicating that at least one of the independent variables significantly impacted the dependent variable. Specifically, each of the main effects for sadness emotion induction [*F* (1, 21,750) = 6.059, *p* < 0.05] is significant. The mean choice value in the sadness group was 1.372, which was significantly lower than that in the neutral group, recorded at 1.403. In our experimental setting, where 1 represents the choice for immediate rewards and 2 indicates the choice for future rewards, these results affirm that the induction of sadness emotion had a discernible impact on participants’ intertemporal choice behavior, leading them to exhibit a greater preference for immediate rewards. This finding supports the establishment of research hypothesis 1. Then the concomitant variables like gender [*F* (1, 21,750) = 49.724, *p* < 0.001], money [*F* (1, 21,750) = 1332.582, *p* < 0.001], degree [*F* (1, 21,750) = 21.007, *p* < 0.001], and interval [*F* (1, 21,750) = 93.214, *p* < 0.001] reached significance. This suggests that emotion induction, gender, baseline money, task difficult degree, and intertemporal intervals significantly influenced participants’ intertemporal choice behavior.

**Table 3:** Results of covariance analysis.

Source	Type III SS	df	MS	*F*	Sig.
Corrected model	340.952	9	37.884	171.214	0.000
Intercept	649.976	1	649.976	2937.545	0.000
Emotion	1.341	1	1.341	6.059	0.014
Gender	11.002	1	11.002	49.724	0.000
Money	294.854	1	294.854	1332.582	0.000
Degree	4.648	1	4.648	21.007	0.000
Interval	20.625	1	20.625	93.214	0.000
Emotion * gender	0.205	1	0.205	0.925	0.336
Emotion * money	1.189	1	1.189	5.376	0.020
Emotion * degree	0.114	1	0.114	0.516	0.473
Emotion * interval	0.137	1	0.137	0.620	0.431
Error	4812.513	21,750	0.221		
Total	46909.000	21,760			
Corrected total	5153.465	21,759			

R Squared = 0.168 (Adjusted R Squared = 0.168).

The interaction term of emotion and money was found to be statistically significant [*F* (1, 21,750) = 5.376, *p* < 0.005], indicating that the influence of emotion induction on intertemporal choice behavior varied significantly depending on the level of monetary considerations. But the interaction terms of emotion and gender [*F* (1, 21,750) = 0.925, *p* = 0.336], emotion and degree [*F* (1, 21,750) = 0.516, *p* = 0.473] emotion and interval [*F* (1, 21,750) = 0.620, *p* = 0.431] were not significant. These findings suggest that the influence of sadness emotion induction on intertemporal choice behavior did not significantly vary based on gender, task difficulty, or intertemporal intervals, except baseline money for today, which supported the establishment of hypothesis 2 and the rejection of hypothesis 3 and 4.

The significant main effects of emotion, gender, money, degree, and interval on intertemporal choice behavior offer valuable insights into the factors shaping individuals’ decisions regarding immediate versus delayed rewards. Emotion induction, particularly, emerged as a significant predictor of intertemporal choice, with sadness induction leading to a greater preference for immediate rewards, consistent with prior research highlighting the impact of emotional states on decision-making processes. Moreover, the significant main effect of task difficulty suggests that individuals are more inclined to choose future rewards in tasks with higher difference rates, underscoring the role of cognitive processes in intertemporal decision-making. Gender differences were also observed, with females exhibiting a stronger preference for immediate rewards compared to males, possibly influenced by socio-cultural factors. Additionally, the significant main effect of intertemporal length underscores the importance of time horizons in shaping intertemporal choice preferences. Individuals tend to display weaker preferences for future rewards when presented with longer time intervals, aligning with the temporal discounting phenomenon commonly observed in decision-making contexts.

### 3.3 Estimation of temporal discounting rate

The results of the covariance analysis revealed the impact of interventions targeting sad emotions on participants’ intertemporal decision-making outcomes. To further explore the influence of interventions targeting sad emotions on participants’ intertemporal decision-making preferences, we estimated participants’ discount rates using the hyperbolic function model V=A/(1+kD) (Green and Myerson, 2004; Fellows and Farah, 2005), *A* represents future rewards, *V* represents the present value of rewards in the future, and *k* represents the discount rate. A higher value of *k* indicates a steeper discounting curve, indicating that participants are more inclined to choose immediate rewards.

**Figure 2:**
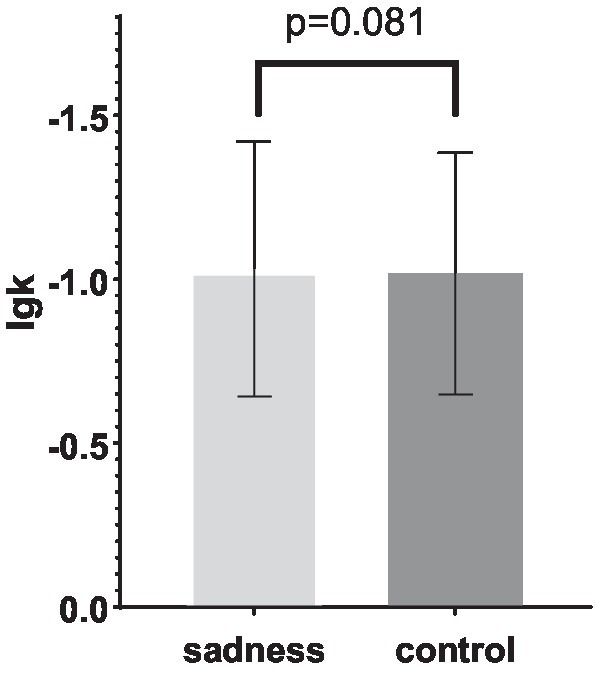
Comparison of logarithmic discounted values of hyperbolic functions in the experimental groups.

The experimental data of each participant in all experimental groups were separately subjected to fitting tests using the hyperbolic, employing nonlinear least squares estimation (Sellitto et al., 2010). To be more precise, *k* is estimated by minimizing the squared deviation between the observed and theoretical values, i.e., \hat{k} = argmin_k sum_{i=1}^N (V_i-A_i/(1+kD_i))^2. We use standard non-linear optimizers in SPSS to compute \hat{k} (alongside its accompanying statistics). The goodness of fit for hyperbolic function, R-squared values is 0.5142, which is lower than previous study (Sellitto et al., 2010). Before conducting the analysis of differences in delay discount rates among different experimental groups, it was necessary to logarithmically transform the rates to meet the assumptions of statistical tests. The results of the difference test for logarithmically transformed discount rates are shown in Figure 2.

For both experimental groups, the logarithmically transformed k value of the sad intervention group was not significantly lower than that of the control group (−1.0317 vs. −1.0181, *p* = 0.081), suggesting that the sad intervention did not yield a discernible difference in delay discount rates (0.1281 vs. 0.1291). This finding contrasts with the effects of negative emotions on delay discounting reported in prior studies (Guan et al., 2015; Calluso et al., 2021; Suo et al., 2021). However, it is noteworthy that previous analyses did indicate a significantly higher frequency of choosing immediate rewards in the sad intervention group.

### 3.4 Logit regression model analysis

To deepen and refine our understanding of how sadness emotion influences individuals’ intertemporal decision-making quantitatively, we conducted subsequent logistic regression analysis. This allowed for a more precise examination of the magnitude and specificity of the effect. To further quantitatively explain the influence of sadness emotion on participants’ intertemporal choices, we established the following logit regression Model (1) for the binary attribute of the dependent variable:

(1) $$\begin{array}{l} \mathrm{Logit}\left(P\right)=\beta 0+\beta 1^{*}\;\mathrm{emotion}+\beta 2^{*}\;\mathrm{gender}\\ \;\;\;\;\;\;\;\;\;\;\;\;\;\;\;\;\;\;\;\;\;\;+\beta 3^{*}\;\mathrm{Money}+\beta 4^{*}\;\mathrm{degree}+\beta 5^{*}\;\mathrm{interval} \end{array}$$

After considering the interaction effects of other control variables with sadness emotion induction, we expanded the logit Model (1) to logit Model (2):

(2) $$\begin{array}{l} \mathrm{Logit}\left(P\right)=\beta 0+\beta 1^{*}\;\mathrm{emotion}+\beta 2^{*}\;\mathrm{gender}\\ \;\;\;\;\;\;\;\;\;\;\;\;\;\;\;\;\;\;\;\;\;\;+\beta 3^{*}\;\mathrm{Money}+\beta 4^{*}\;\mathrm{degree}+\beta 5^{*}\;\mathrm{interval}\\ \;\;\;\;\;\;\;\;\;\;\;\;\;\;\;\;\;\;\;\;\;\;+\beta 6^{*}\;{\mathrm{emotion}}^{*}\;\mathrm{gender}+\beta 7^{*}\;{\mathrm{emotion}}^{*}\;\mathrm{degree}\\ \;\;\;\;\;\;\;\;\;\;\;\;\;\;\;\;\;\;\;\;\;\;+\beta 8^{*}\;{\mathrm{emotion}}^{*}\;\mathrm{money}+\beta 9^{*}\;{\mathrm{emotion}}^{*}\;\mathrm{interval} \end{array}$$

In the above models, “emotion” represents the emotion induction type, including sadness emotion and neutral emotion. “Gender” indicates the gender of the participants. “Money” represents the current amount scale, divided into 2 RMB and 6 RMB. “Degree” denotes the level of task difficulty. “Interval” indicates the intertemporal length, mainly categorized as 3 days and 7 days. The interaction terms involve multiplying other variables with “emotion” to measure the moderating degree of other variables on the relationship between emotional experience and intertemporal choice.

Using the experimental data, parameter estimation is conducted for Models (1) and (2), as shown in Tables 4, 5. Prior to conducting logistic regression analysis, we thoroughly examined the independence of participants and the collinearity among variables. In terms of participant independence, we ensured randomization during recruitment and grouping, as well as maintaining separation throughout the experimental process. These measures were implemented to minimize potential interparticipant influences, thereby supporting the assumption of participant independence. Additionally, we conducted collinearity tests using linear regression (see Table A2). The variance inflation factor (VIF) values between significant variables were all less than 2, indicating the absence of substantial collinearity. These preliminary assessments provide a fundamental basis for conducting logistic regression analysis.

**Table 4:** Logistics regression result for model 1.

Variables	*B*	S.E.	Wald	df	*p*	Exp(B)	95% C.I. for EXP(B)	
							Lower	Upper
Emotion (1)	−0.199	0.03	42.911	1	0.000	0.82	0.772	0.87
Gender (1)	0.266	0.037	52.057	1	0.000	1.305	1.214	1.402
Money	0.256	0.007	1236.055	1	0.000	1.291	1.273	1.31
Degree (1)	−0.132	0.029	20.873	1	0.000	0.877	0.828	0.928
Interval	−0.07	0.007	93.932	1	0.000	0.932	0.919	0.946
Constant	−1.145	0.094	149.981	1	0.000	0.318		

**Table 5:** Logistics regression result for model 2.

Variables	*B*	S.E.	Wald	df	*p*	Exp(B)	95%C.I. for EXP(B)	
							Lower	Upper
Emotion (1)	−2.925	0.205	204.422	1	0.000	0.054	0.036	0.08
Gender (1)	0.097	0.120	0.659	1	0.417	1.102	0.872	1.393
Money	0.301	0.008	1400.065	1	0.000	1.351	1.330	1.373
Degree (1)	−0.410	0.099	17.103	1	0.000	0.663	0.546	0.806
Interval	−0.084	0.008	113.065	1	0.000	0.92	0.906	0.934
Emotion*gender	0.162	0.09	3.247	1	0.072	1.176	0.986	1.402
Emotion*degree	0.274	0.064	18.043	1	0.562	1.315	1.159	1.492
Emotion*money	0.130	0.005	599.506	1	0.000	1.139	1.127	1.151
Emotion*interval	0.270	0.005	2510.88	1	0.147	1.310	1.296	1.324
Constant	−3.627	0.460	62.043	1	0.000	0.027		

For Model (1), all variables exhibit statistically significant parameters. Holding other factors constant, participants are 0.82 times more likely to opt for intertemporal choices following sadness emotion induction compared to neutral emotion, supporting the hypothesis posited in prior research (Lerner et al., 2013). Notably, Participants encountering easy task are 0.877 times likely to favor future rewards compared to those exposed to difficult task, in line with both intuitive expectations and previous studies. Furthermore, an increase in baseline amount (from 2 RMB to 6 RMB) leads to a 1.29 times rise in the occurrence rate of intertemporal choices. Conversely, increasing intertemporal length (from 3 days to 7 days) results in a 0.932 times of the likelihood to select delay rewards over immediate rewards. Lastly, the significant gender parameters reveal that male participants exhibit a stronger preference for immediate rewards than females, with the occurrence rate of choosing future rewards being 1.305 times that of selecting current rewards when transitioning from male to female.

In Model (2), in addition to the variables in Model (1), the analysis explores interaction effects between sadness emotion induction and other variables. Notably, the interaction effect primarily stems from the baseline amount incentive. As the baseline amount increases from 2 RMB to 6 RMB, the likelihood of sadness emotion affecting intertemporal choice decreases by 0.139 times, supporting the acceptance of research hypothesis 2. However, the lack of significant interaction effects with gender, task difficulty, and intertemporal time warrants further investigation and discussion.

## 4 Discussion

This study explores the behavioral differences in intertemporal choice under the influence of specific negative emotions, focusing on sadness compared to neutral emotions. Experimental findings indicate that individuals experiencing sadness exhibit a preference for immediate rewards and demonstrate a stronger present bias. It is important to note that this influence primarily manifests in the frequency of participants selecting immediate rewards, rather than affecting the time discount rate. The study confirms the main effect of sadness on present bias among Chinese participants, prompting further inquiry into the underlying mechanisms driving this influence.

Johnson and Tversky (1983) conducted experimental research demonstrating that inducing sadness in participants through exposure to tragic events increased their perception of various risks and unexpected events. This finding contributed to the development of the “affective generalization hypothesis,” which suggests that exposure to negative emotional stimuli leads individuals to perceive unrelated risk events with the same emotional valence as having an increased likelihood of occurrence. Conversely, positive emotions tend to result in a decreased perception of risk events. Berns et al. (2007) further emphasize that the ability to delay gratification and prioritize future benefits over immediate rewards requires individuals to overcome a sense of urgency, a trait often associated with positive emotions. Research by Ifcher and Zarghamee (2011) and Jin and Isen (2011) supports this notion, showing that positive emotions enhance cognitive flexibility and promote long-term thinking. In contrast, negative emotions, as posited by the valence theory of emotions (Forgas, 1995; Desteno et al., 2000), tend to diminish patience and increase impulsivity. Studies by Rounds et al. (2007) and She et al. (2016) illustrate this phenomenon, indicating that individuals experiencing negative emotions, such as fear or social anxiety, exhibit higher levels of delay discounting and are more prone to short-sighted decision-making.

Similarly, research on depression by Takahashi et al. (2008a,b) suggests a positive correlation between the severity of depressive symptoms and impulsivity, further supporting the notion that negative emotions lead to a heightened focus on immediate gratification. Herman et al. (2003) discusses the concept of a “motivational shift” induced by negative emotions, wherein the desire for immediate gratification outweighs the attractiveness of long-term goals. Empirical evidence by Dorison et al. (2020b) and Lerner et al. (2013) corroborates these findings, demonstrating that individuals experiencing sadness exhibit impatience and a preference for immediate rewards. This study aligns with previous research, confirming that sadness influences intertemporal choice by promoting impulsive decision-making and a focus on short-term outcomes.

The experience of sadness can significantly impact individuals’ mental representations of future events, as suggested by the Construal Level Theory (CLT). According to the CLT, emotions like sadness can influence the construal level, which refers to the mental distance at which events are represented. Sadness and anxiety, for instance, may lead to different construal levels based on the abstract or concrete mental representations of the emotional events (Doré et al., 2015). This aligns with the idea that mental representations of events farther in the future tend to contain fewer sensory and contextual details compared to events closer in time (Gershman and Bhui, 2020). Furthermore, the ability to delay gratification by focusing on future events is facilitated by the right temporoparietal junction, which aids in mental representations of future outcomes (Soutschek et al., 2020). Research also indicates that mental representations of future events are influenced by emotional states. For instance, sad moods have been found to promote self-initiated mental contrasting of future scenarios, aiding in self-regulation and goal commitments (Kappes et al., 2011). Additionally, the study by Shirai and Soshi (2019) suggests that sadness is represented as a specific physical pain through verbal knowledge, indicating an interaction between emotional states and bodily experiences in the representation of sadness. Moreover, the study by Ji et al. (2018) highlights that the emotional consequences of future-oriented mental representations are primarily linked to mental imagery, emphasizing the role of imagination in shaping emotional responses to future events. This is further supported by the findings that participants report greater autonoetic feelings and vivid mental representations when imagining positive and temporally close future events (Lehner and D'Argembeau, 2016).

The findings align with the Appraisal Tendency Framework (ATF), which posits that emotions, including sadness, are accompanied by specific cognitive appraisals that influence decision-making processes (Winterich et al., 2010). The ATF suggests that emotions lead to implicit cognitive predispositions that shape how individuals appraise future events based on the central appraisals characterizing those emotions. This framework emphasizes the role of cognitive appraisals in shaping emotional responses and subsequent decision-making (Winterich et al., 2010). Moreover, research has shown that cognitive appraisals play a crucial role in understanding emotional responses and behavior. Appraisals are linked to emotional experiences, with specific appraisal profiles influencing emotions and decision-making (Zajonc, 1984). The perceived appraisal profiles for different emotions generally align with predictions based on appraisal theory, highlighting the significance of cognitive evaluations in emotional experiences (Laukka and Elfenbein, 2011). Additionally, the relationship between appraisals, emotions, and performance has been observed in various contexts, such as in athletes, where dispositional emotion regulation strategies impact emotional processes and subjective performance (Cece et al., 2021).

In addition to the influence of emotions, various factors contribute to differences in behavioral preferences for intertemporal decisions, including the magnitude of the amount, length of the time period and gender. Our study reveals that as the amount increases, the length of the time period extends, and the difference rate rises, individuals are more likely to choose future rewards. Additionally, male participants exhibit a greater tendency to select future rewards compared to female participants, consistent with previous research findings (Green et al., 1997; Takahashi et al., 2008a,b). However, our study uncovers some inconsistent results at the level of interaction effects. Although the change in the amount is not substantial (from 2 RMB to 6 RMB), it still moderates the preferences of individuals under the influence of sadness. Our literature review suggests that the impact of incentive amounts may be related to the relative gains of the participants, particularly among student participants who may be more sensitive to changes in income levels due to limited fixed income. With an increase in the amount, the influence of sadness diminishes, alleviating the short-sighted behavior of the participants. Even when experiencing sadness, individuals are willing to exhibit more patience in waiting for higher future gains if the possibility of obtaining larger gains exists.

Both the results from the analysis of covariance and the logit regression model consistently indicate that the length of the time period, difference rate, and gender do not significantly moderate the influence of sadness on participants’ current preferences. This lack of significant moderation may be attributed to the relatively small difference in time period lengths, ranging from 3 days to 7 days, which did not induce substantial uncertainty among the participants. Moreover, the characteristics of student participants, who lack a fixed income reference, may have contributed to this lack of significance. Evaluating changes in income uncertainty between two time periods with minor differences is not straightforward, and the influence of emotions further complicates this cognitive process. Similarly, the analysis of different difference rates yields similar results. The discretization of difference rates into high and low categories may have affected individuals’ sensitivity to income variations, making the impact of the difference rate weaker compared to emotions. Consequently, the interaction effect between these two factors may be either weak or significant.

It is essential to note that not all negative emotions lead to this lack of patience in intertemporal choice. Taking into account the valence of emotions and drawing on the Appraisal-Framework Theory, we can explore the impact of different emotions on intertemporal choice based on the six appraisal dimensions of emotions. This approach opens up broad avenues for future research to investigate how different emotions affect intertemporal choice based on these six dimensions (Lerner et al., 2023). Furthermore, the neural mechanisms underlying the influence of emotions on intertemporal choice require further in-depth investigation. Current research on the cognitive processes and neural mechanisms of intertemporal choice has produced different conclusions, such as the Construal Level Theory, Impulsivity, and Self-Control Theory (Berns et al., 2007; Li et al., 2019). Although many studies have identified the involvement of brain areas such as ACC in emotion and decision-making (Kable and Glimcher, 2007; Peters and Büchel, 2010, 2011). At the same time, the difference of neural signals in the intertemporal decision-making process influenced by different negative emotions was also revealed (Suo et al., 2021). But are these differences due to differences in titer between emotions or are there differences in emotion types? Our findings provide experimental insights into the impact of sadness on intertemporal decision-making, but the revelation of the neural mechanism still leaves a lot of room for further research.

A potential limitation of this study lies in the potential confounding influence of cognitive functions, particularly memory load, following the induction of sadness. While the research primarily investigates the impact of sadness on intertemporal decision-making, it is crucial to acknowledge the possibility that observed effects may not solely be driven by changes in mood but also by concurrent cognitive decline. Previous literature indicates that negative mood states can impair cognitive functions such as memory and executive functions (Rusting, 1999; Dobson, 2015; Nusbaum et al., 2018). Thus, it is plausible that the observed alterations in decision-making behavior could be attributed, at least in part, to changes in cognitive processing rather than exclusively to the emotional state induced by the experimental manipulation. To address this potential confound, future studies may consider implementing additional measures to disentangle the effects of mood change from cognitive decline on decision-making.

Another important limitation is that we employed film clips to induce participants’ emotional states, which primarily focused on the type of emotion without considering the valence of the emotion. Recent studies have increasingly utilized the episodic future thinking (EFT), which involves imagining future scenarios, as an emotional intervention to investigate how different types and valences of emotions influence individuals’ intertemporal decision-making (Calluso et al., 2019; Ballance et al., 2022). Positive emotions have been found to decrease individuals’ discount rates, indicating a stronger preference for delayed rewards, while the effects of negative emotions have been less consistent (Suo et al., 2021). Although our study revealed an strong trend to choose immediate reward under the influence of sadness, the lack of control over valence and the arousal effects, associated with our method of emotional induction, somewhat limits the generalizability of our research findings. Finally, in our research design, it is crucial to acknowledge that the interval between the two interperiods is relatively brief. This temporal proximity poses challenges to the efficacy of hyperbolic function fitting for discount rate estimation. Consequently, it diminishes the explanatory capacity regarding the impact of emotions on the outcomes of intertemporal decision-making to a certain degree.

## 5 Conclusion

After inducing feelings of sadness, we focused on examining individuals’ preference behaviors in intertemporal choice under different amounts, difference rates, and time horizons, leading to the following conclusions: Sadness has a significant negative impact on individuals’ intertemporal choice, evoking aversion to uncertainty and reducing patience for waiting, resulting in stronger preference to immediate reward. And the effect gets bigger with the increase in reward scale.

## Data availability statement

The raw data supporting the conclusions of this article will be made available by the authors, without undue reservation.

## Ethics statement

The studies involving humans were approved by Ethics Review Committee of Sichuan Psychological Society. The studies were conducted in accordance with the local legislation and institutional requirements. The participants provided their written informed consent to participate in this study.

## Author contributions

PL: Data curation, Formal analysis, Methodology, Validation, Writing – original draft, Writing – review & editing. HZ: Conceptualization, Funding acquisition, Investigation, Project administration, Resources, Supervision, Writing – original draft, Writing – review & editing. WZ: Conceptualization, Data curation, Investigation, Writing – review & editing. LZ: Data curation, Methodology, Software, Writing – original draft.

## Appendix

**Table A1:** Delay and immediate percentage in each account for all intertemporal tasks.

Delay account	Difference rate	Delay frequency	Delay percentage	Immediate percentage
2.1	5%	124	9.12%	90.88%
2.2	10%	153	11.25%	88.75%
2.3	15%	172	12.65%	87.35%
2.5	25%	265	19.49%	80.51%
2.7	35%	324	23.82%	76.18%
3	50%	499	36.69%	63.31%
3.4	70%	658	48.38%	51.62%
3.9	95%	719	52.87%	47.13%
6.3	5%	209	15.37%	84.63%
6.6	10%	299	21.99%	78.01%
6.9	15%	370	27.21%	72.79%
7.5	25%	667	49.04%	50.96%
8.1	35%	841	61.84%	38.16%
9	50%	903	66.40%	33.60%
10.2	70%	1,080	79.41%	20.59%
11.7	95%	1,100	80.88%	19.12%

**Table A2:** Linear regression result.

Variables	*B*	Std. Error	*t*	Sig.	95.0% CI		Collinearity	
					Lower	Upper	Tolerance	VIF
Constant	1.595	0.023	70.485	0.000	1.551	1.639		
Emotion	−0.045	0.006	−7.257	0.000	−0.057	−0.033	0.896	1.115
Gender	−0.054	0.007	−7.302	0.000	−0.069	−0.040	0.896	1.115
Money	0.058	0.001	39.687	0.000	0.055	0.061	1.000	1.000
Degree	0.352	0.006	59.687	0.000	0.340	0.364	1.000	1.000
Interval	0.015	0.001	10.342	0.000	0.012	0.018	1.000	1.000

Dependent variable: choice.

From the Table A2, the collinearity check result showed that there are limited collinearity between variables.

## References

[ref1] Bahrami R. Borhani K. (2023). Excluded and myopic: Social exclusion increases temporal discounting. PLoS One 18:e0290175. doi: 10.1371/journal.pone.0290175, PMID: 37582119 PMC10426998

[ref2] Ballance B. C. Tuen Y. J. Petrucci A. S. Orwig W. Safi O. K. Madan C. R.. (2022). Imagining emotional events benefits future-oriented decisions. Q. J. Exp. Psychol. 75, 2332–2348. doi: 10.1177/17470218221086637, PMID: 35225089 PMC9619259

[ref3] Berns G. S. Laibson D. Loewenstein G. (2007). Intertemporal choice--toward an integrative framework. Trends Cogn. Sci. 11, 482–488. doi: 10.1016/j.tics.2007.08.011, PMID: 17980645

[ref4] Calluso C. Devetag M. G. Donato C. (2021). “I Feel Therefore I Decide”: Effect of Negative Emotions on Temporal Discounting and Probability Discounting. Brain Sci. 11:1407. doi: 10.3390/brainsci11111407, PMID: 34827406 PMC8615549

[ref5] Calluso C. Tosoni A. Cannito L. Committeri G. (2019). Concreteness and emotional valence of episodic future thinking (EFT) independently affect the dynamics of intertemporal decisions. PLoS One 14:e0217224. doi: 10.1371/journal.pone.0217224, PMID: 31136620 PMC6538244

[ref6] Cece V. Brenas M. Martinent G. (2021). The role of dispositional emotion regulation strategies on the longitudinal emotional process and subjective performance during a competitive season. Eur. J. Sport Sci. 21, 1448–1458. doi: 10.1080/17461391.2020.1862304, PMID: 33295854

[ref8] Desteno D. Petty R. E. Wegener D. T. Rucker D. D. (2000). Beyond valence in the perception of likelihood: the role of emotion specificity. J. Pers. Soc. Psychol. 78, 397–416. doi: 10.1037/0022-3514.78.3.397, PMID: 10743870

[ref9] Dobson M. (2015). Cognitive reactivity to a depressive mood induction procedure across diagnostic categories. J. Depress. Anxiety 4:203. doi: 10.4172/2167-1044.1000203

[ref10] Doré B. Ort L. Braverman O. Ochsner K. (2015). Sadness shifts to anxiety over time and distance from the national tragedy in newtown, connecticut. Psychol. Sci. 26, 363–373. doi: 10.1177/0956797614562218, PMID: 25767209 PMC4398595

[ref12] Dorison C. A. Klusowski J. Han S. Lerner J. S. (2020a). Emotion in organizational judgment and decision making. Organ. Dyn. 49:100702. doi: 10.1016/j.orgdyn.2019.02.004, PMID: 34248771

[ref13] Dorison C. A. Lerner J. S. Heller B. H. Rothman A. J. Kawachi I. I. Wang K.. (2022). In COVID-19 health messaging, loss framing increases anxiety with little-to-no concomitant benefits: Experimental evidence from 84 countries. Affect. Sci. 3, 577–602. doi: 10.1007/s42761-022-00128-3, PMID: 36185503 PMC9510728

[ref14] Dorison C. A. Wang K. Rees V. W. Kawachi I. Ericson K. M. Lerner J. S. (2020b). Sadness, but not all negative emotions, heightens addictive substance use. Proc. Natl. Acad. Sci. 117, 943–949. doi: 10.1073/pnas.1909888116, PMID: 31888990 PMC6969515

[ref15] Dukes D. Abrams K. Adolphs R. Ahmed M. E. Beatty A. Berridge K. C.. (2021). The rise of affectivism. Nat. Hum. Behav. 5, 816–820. doi: 10.1038/s41562-021-01130-8, PMID: 34112980 PMC8319089

[ref16] Elster J. (1998). Emotions and economic theory. J. Econ. Lit. 36, 47–74,

[ref9001] Epper T. Fehr-Duda H. Bruhin A. (2011). Viewing the future through a warped lens: Why uncertainty generates hyperbolic discounting. Journal of risk and uncertainty 43, 169–203.

[ref17] Fang L. Zhaohong Z. Xuejun B. (2009). The duration of happiness and sadness induced by emotional film editing. Stud. Psychol. Behav. 7:32,

[ref18] Fellows L. K. Farah M. J. (2005). Dissociable elements of human foresight: a role for the ventromedial frontal lobes in framing the future, but not in discounting future rewards. Neuropsychologia 43, 1214–1221. doi: 10.1016/j.neuropsychologia.2004.07.018, PMID: 15817179

[ref19] Ferrer R. Ellis E. (2019). Moving beyond categorization to understand affective influences on real world health decisions. Soc. Personal. Psychol. Compass 13:e12502. doi: 10.1111/spc3.12502, PMID: 33912229 PMC8078832

[ref21] Ferrer R. Klein W. Lerner J. Reyna V. Keltner D. (2016). “Emotions and health decision making” in Behavioral economics and public health, 101–132.

[ref22] Forgas J. P. (1995). Emotion and judgment: the affect infusion model (aim). Psychol. Bull. 117, 39–66. doi: 10.1037/0033-2909.117.1.39, PMID: 7870863

[ref23] Gerrards-Hesse A. Spies K. Hesse F. W. (2011). Experimental inductions of emotional states and their effectiveness: a review. Br. J. Psychol. 85, 55–78. doi: 10.1111/j.2044-8295.1994.tb02508.x

[ref24] Gershman S. Bhui R. (2020). Rationally inattentive intertemporal choice. Nat. Commun. 11:3365. doi: 10.1038/s41467-020-16852-y, PMID: 32620804 PMC7335105

[ref25] Gray J. R. (2004). Integration of emotion and cognitive control. Curr. Dir. Psychol. Sci. 13, 46–48. doi: 10.1111/j.0963-7214.2004.00272.x, PMID: 38547188

[ref26] Green L. Myerson J. (2004). A discounting framework for choice with delayed and probabilistic rewards. Psychol. Bull. 130:769. doi: 10.1037/0033-2909.130.5.769, PMID: 15367080 PMC1382186

[ref27] Green L. Myerson J. McFadden E. (1997). Rate of temporal discounting decreases with amount of reward. Mem. Cogn. 25, 715–723. doi: 10.3758/BF03211314, PMID: 9337589

[ref9009] Gross J. J. Levenson R. W. (1995). Emotion elicitation using films. Cognition and Emotion 9, 87–108. doi: 10.1080/02699939508408966

[ref28] Guan S. Cheng L. Fan Y. Li X. (2015). Myopic decisions under negative emotions correlate with altered time perception. Front. Psychol. 6:468. doi: 10.3389/fpsyg.2015.00468, PMID: 25941508 PMC4400848

[ref29] Han D. Duhachek A. Agrawal N. (2014). Emotions shape decisions through construal level: the case of guilt and shame. J. Consum. Res. 41, 1047–1064. doi: 10.1086/678300

[ref31] Herman C. P. Roth D. A. Polivy J. (2003). Effects of the presence of others on food intake: a normative interpretation. Psychol. Bull. 129:873. doi: 10.1037/0033-2909.129.6.873, PMID: 14599286

[ref32] Hirsh J. B. Guindon A. Morisano D. Peterson J. B. (2010). Positive mood effects on delay discounting. Emotion 10:717. doi: 10.1037/a0019466, PMID: 21038955

[ref33] Ifcher J. Zarghamee H. (2011). Happiness and time preference: the effect of positive affect in a random-assignment experiment. Am. Econ. Rev. 101, 3109–3129. doi: 10.1257/aer.101.7.3109

[ref34] Ji J. Holmes E. Mac Leod C. Murphy F. (2018). Spontaneous cognition in dysphoria: reduced positive bias in imagining the future. Psychol. Res. 83, 817–831. doi: 10.1007/s00426-018-1071-y 30097711 PMC6529377

[ref35] Jiang Y. P. Sun H. Y. (2019). Concept, measurements, antecedents and consequences of the effect of emotion on intertemporal choice. Adv. Psychol. Sci. 27, 1622–1630. doi: 10.3724/SP.J.1042.2019.01622

[ref36] Jin S. P. Isen A. M. (2011). Positive affect, intertemporal choice, and levels of thinking: increasing consumers' willingness to wait. J. Mark. Res. 48, 532–543. doi: 10.1509/jmkr.48.3.532

[ref9010] Jin X. Guanghui D. Min J. Guozhi L. (2009). Evaluation of the effect of video materials on emotional arousal. Psychological Exploration 29, 83–87. doi: 10.3969/j.issn.1003-5184.2009.06.017

[ref37] Johnson E. J. Tversky A. (1983). Affect, generalization, and the perception of risk. J. Pers. Soc. Psychol. 45, 20–31. doi: 10.1037/0022-3514.45.1.20, PMID: 38549052

[ref38] Kable J. W. Glimcher P. W. (2007). The neural correlates of subjective value during intertemporal choice. Nat. Neurosci. 10, 1625–1633. doi: 10.1038/nn2007, PMID: 17982449 PMC2845395

[ref39] Kappes H. Oettingen G. Mayer D. Maglio S. (2011). Sad mood promotes self-initiated mental contrasting of future and reality. Emotion 11, 1206–1222. doi: 10.1037/a0023983, PMID: 21707148

[ref40] Keidel K. Rramani Q. Weber B. Murawski C. Ettinger U. (2021). Individual differences in intertemporal choice. Front. Psychol. 12:643670. doi: 10.3389/fpsyg.2021.643670 33935897 PMC8085593

[ref41] Laukka P. Elfenbein H. (2011). Emotion appraisal dimensions can be inferred from vocal expressions. Soc. Psychol. Personal. Sci. 3, 529–536. doi: 10.1177/1948550611428011 PMC5717659 29291085

[ref42] Lazarus R. S. (1991). Progress on a cognitive-motivational-relational theory of emotion. Am. Psychol. 46:819. doi: 10.1037/0003-066X.46.8.819, PMID: 1928936

[ref43] Lehner E. D'Argembeau A. (2016). The role of personal goals in autonoetic experience when imagining future events. Conscious. Cogn. 42, 267–276. doi: 10.1016/j.concog.2016.04.002, PMID: 27089529

[ref44] Lempert K. M. Glimcher P. W. Phelps E. A. (2015). Emotional arousal and discount rate in intertemporal choice are reference dependent. J. Exp. Psychol. Gen. 144, 366–373. doi: 10.1037/xge0000047, PMID: 25602754 PMC4388786

[ref45] Lerner J. S. Dorison C. Kim J. (2023). How do emotions affect decision making? doi: 10.31234/osf.io/xbsza,

[ref46] Lerner J. S. Gonzalez R. M. Small D. A. Fischhoff B. (2003). Effects of fear and anger on perceived risks of terrorism: A national field experiment. Psychol. Sci. 14, 144–150. doi: 10.1111/1467-9280.01433 12661676

[ref47] Lerner J. S. Keltner D. (2000). Beyond valence: Toward a model of emotion-specific influences on judgment and choice. Cognit. Emot. 14, 473–493. doi: 10.1080/026999300402763

[ref48] Lerner J. S. Keltner D. (2001). Fear, anger, and, risk. J. Pers. Soc. Psychol. 81:146. doi: 10.1037/0022-3514.81.1.146 11474720

[ref49] Lerner J. S. Li Y. Valdesolo P. Kassam K. S. (2015). Emotion and decision making. Annu. Rev. Psychol. 66, 1–33. doi: 10.1146/annurev-psych-010213-115043 25251484

[ref50] Lerner J. S. Li Y. Weber E. U. (2013). The financial costs of sadness. Psychol. Sci. 24, 72–79. doi: 10.1177/0956797612450302, PMID: 23150274

[ref51] Lerner J. S. Small D. A. Loewenstein G. (2004). Heart strings and purse strings: carryover effects of emotions on economic decisions. Psychol. Sci. 15:337. doi: 10.1111/j.0956-7976.2004.00679.x, PMID: 15102144

[ref52] Li H. Guo Y. Yu Q. (2019). Self-control makes the difference: the psychological mechanism of dual processing model on internet addicts’ unusual behavior in intertemporal choice. Comput. Hum. Behav. 101, 95–103. doi: 10.1016/j.chb.2019.07.010

[ref53] Li X. M. Xie J. (2012). The influence mechanism of incidental emotions on choice deferral. Acta Psychol. Sin. 44, 1641–1650. doi: 10.3724/SP.J.1041.2012.01641

[ref9002] Li A. Peng Y. Xiong G. (2015). Are Pregnant Women More Foresighted? The Effect of Pregnancy on Intertemporal Choice. Acta Psychologica Sinica 47:1360.

[ref57] Luo S. Ainslie G. Monterosso J. (2014). The behavioral and neural effect of emotional primes on intertemporal decisions. Soc. Cogn. Affect. Neurosci. 9, 283–291. doi: 10.1093/scan/nss132, PMID: 23160811 PMC3980799

[ref59] McClure S. M. Laibson D. I. Loewenstein G. Cohen J. D. (2004). Separate neural systems value immediate and delayed monetary rewards. Science 306, 503–507. doi: 10.1126/science.1100907, PMID: 15486304

[ref60] Molouki S. Hardisty D. J. Caruso E. M. (2019). The sign effect in past and future discounting. Psychol. Sci. 30, 1674–1695. doi: 10.1177/0956797619876982, PMID: 31674883

[ref61] Nusbaum A. T. Wilson C. G. Stenson A. Hinson J. M. Whitney P. (2018). Induced positive mood and cognitive flexibility: evidence from task switching and reversal learning. Collabra Psychol. 4:25. doi: 10.1525/collabra.150

[ref9003] Patak M. Reynolds B. (2007). Question-based assessments of delay discounting: do respondents spontaneously incorporate uncertainty into their valuations for delayed rewards? Addictive behaviors 32, 351–357. 16647214 10.1016/j.addbeh.2006.03.034

[ref62] Peters J. Büchel C. (2010). Episodic future thinking reduces reward delay discounting through an enhancement of prefrontal-mediotemporal interactions. Neuron 66, 138–148. doi: 10.1016/j.neuron.2010.03.026, PMID: 20399735

[ref63] Peters J. Büchel C. (2011). The neural mechanisms of inter-temporal decision-making: understanding variability. Trends Cogn. Sci. (Regul. Ed.) 15, 227–239. doi: 10.1016/j.tics.2011.03.002 21497544

[ref64] Raeva D. Mittone L. Schwarzbach J. (2010). Regret now, take it now: On the role of experienced regret on intertemporal choice. J. Econ. Psychol. 31, 634–642. doi: 10.1016/j.joep.2010.04.006

[ref65] Rounds J. S. Beck J. G. Grant D. M. (2007). Is the delay discounting paradigm useful in understanding social anxiety? Behav. Res. Ther. 45, 729–735. doi: 10.1016/j.brat.2006.06.007, PMID: 16890909

[ref66] Rusting C. (1999). Interactive effects of personality and mood on emotion-congruent memory and judgment. J. Pers. Soc. Psychol. 77, 1073–1086. doi: 10.1037/0022-3514.77.5.1073, PMID: 10573881

[ref68] Seaman K. L. Abiodun S. J. Fenn Z. Samanez-Larkin G. R. Mata R. (2022). Temporal discounting across adulthood: A systematic review and meta-analysis. Psychol. Aging 37, 111–124. doi: 10.1037/pag0000634, PMID: 35113618 PMC8827494

[ref69] Sellitto M. Ciaramelli E. di Pellegrino G. (2010). Myopic discounting of future rewards after medial orbitofrontal damage in humans. J. Neurosci. 30, 16429–16436. doi: 10.1523/JNEUROSCI.2516-10.2010, PMID: 21147982 PMC6634874

[ref70] She S. X. Zheng X. W. Zhou J. Yang S. S. (2016). Does fear reduce the patience of intertemporal selection?-- Evidence from behavioral experiments. Psychol. Explor. 36, 25–30. doi: 10.3969/j.issn.1003-5184.2016.01.005

[ref71] Shirai M. Soshi T. (2019). Why is heartache associated with sadness? sadness is represented by specific physical pain through verbal knowledge. PLoS One 14:e0216331. doi: 10.1371/journal.pone.0216331, PMID: 31042783 PMC6493756

[ref9005] Small D. A. Lerner J. S. (2008). Emotional policy: Personal sadness and anger shape judgments about a welfare case. Political psychology 29, 149–168.

[ref72] Soutschek A. Moisa M. Ruff C. Tobler P. (2020). The right temporoparietal junction enables delay of gratification by allowing decision makers to focus on future events. PLoS Biol. 18:e3000800. doi: 10.1371/journal.pbio.3000800, PMID: 32776945 PMC7447039

[ref73] Stillman P. Lee H. Deng X. Unnava H. Cunningham W. Fujita K. (2017). Neurological evidence for the role of construal level in future-directed thought. Soc. Cogn. Affect. Neurosci. 12, 937–947. doi: 10.1093/scan/nsx022, PMID: 28338716 PMC5472149

[ref74] Suo T. Jia X. Song X. Liu L. (2021). The differential effects of anger and sadness on intertemporal choice: An ERP study. Front. Neurosci. 15:638989. doi: 10.3389/fnins.2021.638989, PMID: 34305513 PMC8296139

[ref9008] Takahashi T. Hadzibeganovic T. Cannas S. A. Makino T. Fukui H. Kitayama S. (2010). Cultural neuroeconomics of intertemporal choice. Journal of Behavioral Economics and Finance 3, 133–135. 19675524

[ref75] Takahashi T. Oono H. Inoue T. Boku S. Kako Y. Kitaichi Y.. (2008a). Depressive patients are more impulsive and inconsistent in intertemporal choice behavior for monetary gain and loss than healthy subjects-an analysis based on tsallis' statistics. Neuro Endocrinol. Lett. 29, 351–358, PMID: 18580849

[ref76] Takahashi T. Oono H. Radford M. H. (2008b). Psychophysics of time perception and intertemporal choice models. Phys. A Stat. Mech. Appl. 387, 2066–2074. doi: 10.1016/j.physa.2007.11.047, PMID: 28130716

[ref77] Trope Y. Liberman N. (2000). Temporal construal and time-dependent changes in preference. J. Pers. Soc. Psychol. 79, 876–889. doi: 10.1037/0022-3514.79.6.876, PMID: 11138758

[ref78] Trope Y. Liberman N. (2003). Temporal construal. Psychol. Rev. 110, 403–421. doi: 10.1037/0033-295X.110.3.403, PMID: 12885109

[ref79] Wang P. Liu Y. F. (2009). The effect of mood on intertemporal choice. Journal of Psychological Science 32, 1318–1320.

[ref80] Winterich K. Han S. Lerner J. (2010). Now that i’m sad, it’s hard to be mad: the role of cognitive appraisals in emotional blunting. Personal. Soc. Psychol. Bull. 36, 1467–1483. doi: 10.1177/0146167210384710, PMID: 20876386

[ref85] Zajonc R. (1984). On the primacy of affect. Am. Psychol. 39, 117–123. doi: 10.1037/0003-066X.39.2.117, PMID: 38564245

[ref88] Zhou L. Yang Y. Li S. (2022). Music-induced emotions influence intertemporal decision making. Cognit. Emot. 36, 211–229. doi: 10.1080/02699931.2021.1995331, PMID: 34702138

